# Computer-Based Identification of Potential Druggable Targets in Multidrug-Resistant *Acinetobacter baumannii*: A Combined In Silico, In Vitro and In Vivo Study

**DOI:** 10.3390/microorganisms10101973

**Published:** 2022-10-05

**Authors:** Omar H. Badie, Ahmed F. Basyony, Reham Samir

**Affiliations:** 1 Department of Microbiology and Immunology, Faculty of Pharmacy, Egyptian Russian University, Cairo 11829, Egypt; 2 Department of Microbiology and Immunology, Faculty of Pharmacy, Cairo University, Cairo 11562, Egypt

**Keywords:** *Acinetobacter baumannii*, multidrug-resistant, subtractive proteomics, drug targets, aspartate 1-decarboxylase, malonic acid, D-tartaric acid, citric acid, wound infection

## Abstract

The remarkable rise in antimicrobial resistance is alarming for *Acinetobacter baumannii*, which necessitates effective strategies for the discovery of promising anti-acinetobacter agents. We used a subtractive proteomics approach to identify unique protein drug targets. Shortlisted targets passed through subtractive channels, including essentiality, non-homology to the human proteome, druggability, sub-cellular localization prediction and conservation. Sixty-eight drug targets were shortlisted; among these, glutamine synthetase, dihydrodipicolinate reductase, UDP-N-acetylglucosamine acyltransferase, aspartate 1-decarboxylase and bifunctional UDP-N-acetylglucosamine diphosphorylase/glucosamine-1-phosphate N-acetyltransferase were evaluated in vitro by determining the minimum inhibitory concentration (MIC) of candidate ligands, citric acid, dipicolinic acid, D-tartaric acid, malonic acid and 2-(N-morpholino)ethanesulfonic acid (MES), respectively, which ranged from 325 to 1500 μg/mL except for MES (25 mg/mL). The candidate ligands, citric acid, D-tartaric acid and malonic acid, showed good binding energy scores to their targets upon applying molecular docking, in addition to a significant reduction in *A. baumannii* microbial load in the wound infection mouse model. These ligands also exhibited good tolerability to human skin fibroblast. The significant increase in the MIC of malonic acid in β-alanine and pantothenate-supplemented media confirmed its selective inhibition to aspartate 1-decarboxylase. In conclusion, three out of sixty-eight potential *A. baumannii* drug targets were effectively inhibited in vitro and in vivo by promising ligands.

## 1. Introduction

*Acinetobacter baumannii*, which belongs to the most serious multidrug-resistant (MDR) ESKAPE pathogens, including *Enterococcus faecium*, *Staphylococcus aureus*, *Klebsiella pneumoniae*, *Acinetobacter baumannii*, *Pseudomonas aeruginosa* and *Enterobacter* spp., has globally acquired the attention of the medical field as a public health threat. This attention is due to its ability to cause nosocomial infection, especially in intensive care units (ICU), and to develop multiple resistance mechanisms [1]. It is involved in a number of hospital-acquired infections including pneumonia, bacteremia, urinary tract infections, meningitis and wound infections [2]. Unfortunately, MDR *A. baumannii* is usually related to prolonged hospital accommodation, a high possibility of ICU admission and high morbidity and mortality rates [3]. Widespread MDR *A. baumannii* has been observed along with the availability of many classes of antibiotics to which *A. baumannii* has shown resistance [4]. This case prompted the World Health Organization (WHO) to include *A. baumannii* in the list of the antibiotic-resistant priority pathogens, categorizing it according to the urgency of the need for new antibiotics as “critical” [5]. In addition, the Centers for Disease Control and Prevention (CDC) has supported focusing on drug discovery for infections of the highest need for those caused by *A. baumannii*, considering it as an “urgent threat” [6]. 

The emerging multi-drug resistance and the deficiency of suitable antibiotics have demanded a search for novel antibacterial agents. Traditionally, the development of antibacterial agents depends on the screening of compounds, mostly natural ones, against different bacteria. This approach is used together with the chemical modification of already existing compounds with antibacterial activity [7,8]. 

This conventional approach, used for the discovery and development of antibacterial agents, involves expensive, time-consuming and laborious techniques and also yields fewer drug targets. On the other hand, computational approaches result in a transformation in the process of drug discovery through more simple means of identification of drug targets [9]. The identification of high-value bacterial targets is the starting point in the process of drug discovery [7]. This is based on the utilization of vast available OMICS datasets of different microbes, which offer an increased number of opportunities for drug discovery against resistant pathogens. Subtractive proteomics, metabolic pathways analysis and structural bioinformatics approaches are now employed for the development of new drugs and combating antimicrobial resistance, acting as a complement to conventional approaches [10]. 

Subtractive proteomics, the approach used in the current study, allows for the selection of vital proteins related to the pathogen while absent in the host, followed by checking the presence of drug molecules with possible binding affinity to these proteins in a channel known as “druggability”. These proteins are then regarded as putative drug targets [10]. These drug targets are also checked for cytoplasmic localization in addition to conservation to ensure their use in the control of multiple strains of *A. baumannii*.

Using the subtractive proteomics approach, potential drug targets were previously identified in several other pathogens, including *Escherichia coli*, *Helicobacter pylori*, *Mycobacterium avium*, *Pseudomonas aeruginosa*, *Shigella flexneri*, *Staphylococcus aureus*, *Staphylococcus saprophyticus*, *Stenotrophomonas maltophilia* and *Streptococcus pneumoniae* [11,12,13,14,15,16,17,18,19]. Although potential drug targets in *A. baumannii* have been identified in former studies [20,21,22], the current study proceeds to further validate the data obtained by the in silico approach. This validation was performed by in vitro and in vivo testing to evaluate the antibacterial activity of selected drugs against *A. baumannii* ATCC 19606.

## 2. Materials and Methods

The proteome of *A. baumannii* was analyzed to identify potential druggable targets. The subtractive proteomics approach was used to reveal these targets.

### 2.1. Retrieval of Essential Druggable Proteins Using the In Silico Approach

#### 2.1.1. Retrieval of Essential Proteins

The sequences of the essential proteins of *A. baumannii* ATCC 17978 were downloaded from the Online GEne Essentiality (OGEE) database (available at https://v3.ogee.info/, accessed on 3 July 2020) with their corresponding identification numbers (Table S1). The OGEE contains lists of essential genes with their corresponding sequences and other features [23].

#### 2.1.2. Identification of Human Non-Homologous Proteins

These essential protein sequences were aligned against a non-redundant database of *Homo sapiens* (taxid:9606) with the NCBI Protein Basic Local Alignment Search Tool (BLASTp) (https://blast.ncbi.nlm.nih.gov/, accessed on 25 July 2020) with an estimated expected threshold value of 0.0005, a word size of 6 and the BLOSUM62 matrix. After the analysis, only proteins that showed no homology with the human proteome passed to the next step. Proteins having any significant alignment score (even if <40) with the human proteome were removed to avoid any possible harmful effects on human-similar proteins.

#### 2.1.3. Druggability Analysis

The potential proteins were analyzed to find promising ligands in the DrugBank database version 5.1.7 [24]. The BLASTp of proteins was performed with default parameters against a list of compounds found within the DrugBank, with an expected threshold of 0.00001 and the drug type filter set to include all drug types. This database comprises comprehensive information on drugs and drug targets. It contains a variety of drugs consisting of FDA-approved drugs, experimental drugs and nutraceuticals [25].

#### 2.1.4. Sub-Cellular Localization Prediction 

The sub-cellular localization of the retrieved proteins was predicted through the well-known tool PSORTb version 3.0.3 (available at https://www.psort.org/psortb/, accessed on 14 August 2020) [26]. It detects the compartment to which a protein belongs, such as the cytoplasmic membrane, cytoplasm, cell wall and extracellular. Proteins that were predicted as cytoplasmic were those of interest for drug development and so were included in the subsequent steps, and others were excluded. Proteins with unknown subcellular localization were predicted through another subcellular localization predictor CELLO v.2.5 (available at http://cello.life.nctu.edu.tw/, accessed on 16 August 2020) [27].

#### 2.1.5. Conservation of Potential Proteins in *A. baumannii*

The retrieved cytoplasmic protein targets were checked for conservation in each of the 121 available strains of *A. baumannii* (after removal of repetitions) in the NCBI database (Table S2) using the NCBI BLASTp tool, with an expected threshold of 0.0005. Conserved proteins with >200 alignment scores in all tested *A. baumannii* strains passed to the next step.

#### 2.1.6. Comparison of Shortlisted Proteins to the *A. baumannii* ATCC 19606 Proteome

*A. baumannii* ATCC 19606 was used for further in vitro and in vivo testing in the current study. Therefore, the alignment of the potential proteins was performed against the proteome of *A. baumannii* ATCC 19606 using the NCBI BLASTp tool with an expected threshold of 0.0005. Any protein that showed a percentage identity of <95% was excluded.

#### 2.1.7. Detection of the Pathways Involving the Shortlisted Proteins 

To identify the pathways in which each protein is involved, the Kyoto Encyclopedia of Genes and Genomes (KEGG) database (available at https://www.genome.jp/kegg/) was used [28,29,30].

### 2.2. Testing of the In Vitro Antimicrobial Activity of Candidate Ligands against A. baumannii ATCC 19606 

The testing of in vitro antimicrobial activity of candidate ligands involved the determination of the minimum inhibitory concentration (MIC) of five ligands, citric acid, dipicolinic acid, D-tartaric acid, malonic acid and 2-(N-morpholino)ethanesulfonic acid (MES), against *A. baumannii* ATCC 19606.

#### 2.2.1. Preparation of the Ligand Solutions

The solutions of candidate ligands were prepared by dissolving citric acid (Loba Chemie, Mumbai, India), dipicolinic acid (Alfa Aesar, Karlsruhe, Germany), D-tartaric acid (SigmaAldrich, Hamburg, Germany), malonic acid (Loba Chemie, Mumbai, India) and MES (Caisson Laboratories, Smithfield, UT, USA) in distilled water, and they were sterilized by membrane filtration using a 0.22 μm-pore-size syringe filter (StarTech, Northampton, UK). The tested concentrations of citric acid, D-tartaric acid and malonic acid each varied from 6.25 mg/mL to 0.09 mg/mL. Being less soluble in water, the tested concentrations of dipicolinic acid varied from 1.5 mg/mL to 0.09 mg/mL. Moreover, the tested concentrations of MES ranged from 50 mg/mL to 0.78 mg/mL. 

#### 2.2.2. Culturing of the Bacterial Strain and Inoculum Preparation

*A. baumannii* ATCC 19606 was cultured according to the Clinical and Laboratory Standards Institute (CLSI) on tryptic soy agar (Lab M, Heywood, UK) and was incubated at 37 °C for 18–24 h under aerobic conditions [31]. For the inoculum preparation, isolated colonies of *A. baumannii* were suspended in sterile saline, and the suspension was adjusted to reach a turbidity equivalent to a 0.5 McFarland standard, containing approximately 1 × 10^8^ CFU/mL [31].

#### 2.2.3. Determination of the MIC of Candidate Ligands 

The MIC was determined using the broth micro-dilution method according to the CLSI M07-A9 guidelines [31]. The procedure was performed using 96-well microtiter plates with round bottoms. Each well was primarily filled with 100 μL of Mueller–Hinton broth (Oxoid, Cheshire, UK). From the stock solution of each ligand (with a concentration = 12.5 mg/mL, except for dipicolinic acid and MES, with concentrations = 3 mg/mL and 100 mg/mL, respectively), 100 μL was serially diluted in the broth in order to test their previously mentioned concentrations. The previously prepared inoculum was then diluted 1:20 followed by the addition of 10 μL of the diluted inoculum to each well for the final tested concentration of bacteria to be approximately 5 × 10^5^ CFU/mL. Each experiment was carried out in triplicate. The well of the lowest concentration that showed complete inhibition of visible growth after incubation aerobically at 37 °C for 18–24 h, represented the MIC of that ligand.

### 2.3. Molecular Docking of Candidate Ligands to Their Target Proteins

The candidate ligands (citric acid, D-tartaric acid and malonic acid) that showed the best MIC values proceeded to the next testing steps, starting with molecular docking.

All the molecular modeling studies were performed using the Molecular Operating Environment (MOE, 2019.0102) software. All minimizations were carried out with MOE until an RMSD gradient reached 0.1 kcal∙mol^−1^Å^−1^ with an MMFF94x force field, and the partial charges were automatically calculated.

The X-ray crystallographic structure of glutamine-dependent NAD synthetase from *A. baumannii* in complex with the co-crystalized ligand adenosine diphosphate (ADP), (PDB ID: 5KHA) was downloaded from a protein data bank (https://www.rcsb.org/structure/5KHA, accessed on 18 September 2021). The X-ray crystallographic structure of aspartate decarboxylase in complex with the co-crystalized ligand N~2~-(2-Amino-1-Methyl-2-Oxoethylidene)Asparaginate (NSN), (PDB ID: 1UHE) was downloaded from a protein data bank (https://www.rcsb.org/structure/1UHE, accessed on 18 September 2021). The X-ray crystallographic structure of UDP-N-acetylglucosamine acyltransferase (LpxA) in complex with Uridine-Diphosphate-N-Acetylglucosamine (UD1), (PDB ID: 2JF3) was downloaded from a protein data bank (https://www.rcsb.org/structure/2JF3, accessed on 18 September 2021). 

For every co-crystallized enzyme, water molecules and ligands which were not involved in the binding were removed, and the protein was prepared for the docking study using Protonate 3D protocol in MOE with default options. The co-crystalized ligands (ADP, NSN and UD1) were used to define the binding site for docking. The London dG scoring function and Triangle Matcher placement method were used for docking.

### 2.4. In Vivo Effect of Candidate Ligands on Wound Infection in the Mouse Model

#### 2.4.1. Ethical Statement

The procedures involved in the animal model were performed according to the policies approved by the Research Ethics Committee of the Faculty of Pharmacy, Cairo University, Cairo, Egypt (Approval No. MI-2584), following the “Guide for the Care and Use of Laboratory Animals” published by the Institute of Laboratory Animal Research (USA).

#### 2.4.2. Induction of Infection

The animal model involved the use of fifty adult male BALB/c mice weighing 25–35 g, kept in laboratory animal housing at the Faculty of Pharmacy, Cairo University. At room temperature with an alternating 12 h light–dark cycle, the mice were supplied with food and water. The mice were observed in the beginning to avoid any sign of skin inflammation.

The procedure was performed as described by Ismail et al. [32], as follows:

Prior to wounding, each mouse was subjected to general anesthesia by intraperitoneal injection of 0.25 mL of 2,2,2-tribromoethanol in 2-Methyl-2-butanol, with a concentration of 25 mg/mL. Back hair was shaved from the cervical to mid-lumbar dorsum using an electric hair clipper, followed by washing the skin with ethanol. A 1 cm × 1 cm full-thickness excisional wound was performed by elevating the shaved skin using forceps and then by cutting the desired part of the skin with scissors. For the induction of infection, *A. baumannii* ATCC 19606 was used to prepare a suspension containing 1 × 10^8^ CFU/mL. A 10 μL inoculum was pipetted over the wound of each mouse and was allowed to be absorbed for 1 min.

#### 2.4.3. Treatment of *A. baumannii*-Infected Wounds by Candidate Ligands

Solutions of candidate ligands (citric acid, D-tartaric acid and malonic acid) were prepared in distilled water and were sterilized by membrane filtration with concentrations equivalent to 10× the MIC of each one. A solution of the positive drug control cefepime was prepared with a concentration of 80 μg/mL, which was equivalent to 10× its MIC against *A. baumannii* [33].

The treatment procedures started 24 h after infection by randomly classifying the mice into five groups, each containing ten mice. Each group received 25 μL from one of the solutions, as follows:

- Group 1: Dulbecco’s Phosphate-buffered saline (PBS) (Biowest, France)
- Group 2: Cefepime solution (Sandoz, Egypt)
- Group 3: Citric acid solution
- Group 4: D-tartaric acid solution
- Group 5: Malonic acid solution

#### 2.4.4. Mice Euthanization and Counting the Colonies on the Wounded Skin

Mice were euthanized, and the wounded skin was removed. After cutting the wounded skin into small parts, it was homogenized in 1 mL PBS using Witeg^®^ HG-15D homogenizer at 1500 rpm until obtaining a homogenous suspension. A viable count technique was then used to determine the number of colony-forming units in the removed wounded skin. From each homogenate, 20 μL aliquots were added on 180 μL of PBS and were then ten-fold serially diluted. From each dilution, 10 μL was dropped on the surface of Luria–Bertani (LB) agar (Lab M, UK). This LB agar was prepared containing 50 µg/mL ampicillin, to which *A. baumannii* ATCC 19606 is known to be resistant [34]. After incubation, colonies were counted, and the viable count was calculated for each mouse.

### 2.5. In Vitro Cytotoxicity Assay of Candidate Ligands against Human Skin Fibroblast

Candidate ligands were tested against Human Skin Fibroblast (HSF) (Nawah Scientific Inc., Cairo, Egypt) to evaluate the potential cytotoxic effects of each of them on mammalian cells in vitro. Cells were maintained in Dulbecco’s modified eagle medium (DMEM) supplemented with 100 units/mL of penicillin, 100 mg/mL of streptomycin and 10% of heat-inactivated fetal bovine serum in a humidified 5% (v/v) CO_2_ atmosphere at 37 °C. The cell viability of HSF was tested by a Sulforhodamine B (SRB) assay. Briefly, aliquots of 100 μL cell suspension (5 × 10^3^ cells) were in 96-well plates and were incubated in complete media for 24 h. The cells were treated with another aliquot of 100 μL media containing ligands at various concentrations equivalent to 5× and 10× the MIC of each one. After 2 h of ligand exposure, the cells were fixed by replacing the media with 150 μL of 10% Trichloroacetic acid (TCA) and were incubated at 4 °C for 1 h. The TCA solution was discarded, and the cells were rinsed five times with distilled water. Aliquots of 70 μL SRB solution (0.4% w/v) were added and incubated at room temperature for 10 min in a dark place. Plates were allowed to air-dry overnight after washing them three times with 1% acetic acid. To dissolve the protein-bound SRB stain, 150 μL of Trisaminomethane (TRIS) (10 mM) was then added; the absorbance was measured at 540 nm using a BMG LABTECH^®^-FLUOstar Omega microplate reader (Ortenberg, Germany) [35,36,37].

### 2.6. In Vitro Confirmation of Aspartate 1-Decarboxylase Inhibition by Malonic Acid

To confirm the selective inhibition of aspartate 1-decarboxylase by malonic acid, the MIC value of malonic acid against *A. baumannii* ATCC 19606 was tested in the presence of β-alanine or pantothenate, the downstream products of the aspartate 1-decarboxylase enzyme [38]. To supply the media with sub-inhibitory concentrations, the MIC values of β-alanine and pantothenate against *A. baumannii* ATCC 19606 were each initially determined by the broth micro-dilution method. The MIC values of malonic acid were then determined after supplementation with a range of sub-inhibitory concentrations of β-alanine or pantothenate, as follows:

First, β-alanine and pantothenate were dissolved in Mueller–Hinton broth to prepare stock solutions with 10× the required concentrations. In a 96-well microtiter plate, aliquots of 90 μL of Mueller–Hinton broth were added to each well in the row, followed by the addition of 90 μL of 12 mg/mL malonic acid that was two-fold serially diluted in each row. From the stock solution of either β-alanine or pantothenate, aliquots of only 10 μL of either β-alanine or pantothenate were added to each well in the row to test the required concentrations (50–0.39 mg/mL) and (40–0.3125 mg/mL), respectively. Furthermore, inocula were prepared and then added with the same volume, as mentioned previously in 2.2.2. The MIC of malonic acid was concurrently determined in the absence of both supplements after the addition of 10 μL of Mueller–Hinton broth instead. All plates were incubated at 37 °C for 18–24 h under aerophilic conditions. The well of the lowest concentration that showed the complete inhibition of visible growth represented the MIC of malonic acid. The experiment was carried out in triplicate. 

### 2.7. Statistical Analysis 

GraphPad Prism 8.0.1 (GraphPad Software, Inc., San Diego, CA, USA) was used to perform a one-way ANOVA followed by Tukey’s multiple comparisons test (*p*-value < 0.05). It was also used for performing a correlation test and for the determination of the Pearson correlation coefficient (r). 

## 3. Results

### 3.1. Retrieval of Essential Druggable Proteins Using the In Silico Approach

**Figure 1:**
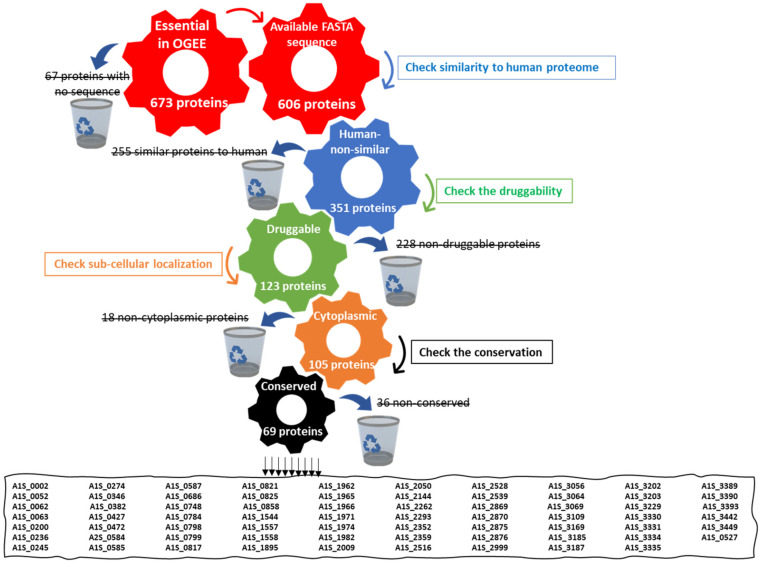
A diagram showing the systemic workflow of drug target identification using a subtractive proteomics approach. The analysis involves passing five channels, using a different tool in each one. The first channel includes the retrieval of essential proteins from the Online GEne Essentiality (OGEE) database. The second one involves checking their homology with a human host. The third channel involves checking their druggability using DrugBank. The fourth one involves the sub-cellular localization of target proteins using PSORTb then CELLO tools. The fifth channel involves checking the conservation of the target proteins in all available *Acinetobacter baumannii* strains using the NCBI BLASTp tool. Finally, the figure shows the 69 shortlisted target proteins with their code in OGEE. The black horizontal lines mean “excluded candidates”.

A total of 673 essential proteins for *A. baumannii* ATCC 17978 were retrieved from the Online GEne Essentiality (OGEE) database. A total of 67 of them had no available sequence in the database. To minimize cross reactivity with human-similar proteins, the BLASTp tool was used, where targets that showed any similarity to *Homo sapiens*, even if minor, were omitted. After the omission of the 255 proteins that showed any significant similarity to *Homo sapiens*, 351 proteins were nominated as essential non-homologous proteins to humans (Figure 1). Searching the druggability of these proteins using DrugBank resulted in 123 proteins with possible ligands. The check of the sub-cellular localization of these proteins using PSORTb revealed that 99 of them were cytoplasmic proteins, 15 belonged to the cytoplasmic membrane and only 9 had unknown localization. Further checks for proteins with unknown subcellular localization using CELLO resulted in the exclusion of 3 non-cytoplasmic proteins for the final count to be 105 cytoplasmic protein targets (Figure 1).

In general, proteins located in the cytoplasm are considered good candidates for drug development, so the 105 cytoplasmic proteins proceeded to the subsequent step. These cytoplasmic proteins were checked for conservation in each of the *A. baumannii* strains that were available in the NCBI database (Table S2). The conservation of proteins ensures the efficacy of candidate drugs against all available *A. baumannii* strains other than that used in the current study, *A. baumannii* ATCC 19606. A total of 69 proteins showed conservation with a significant similarity of >200 alignment scores in all the available *A. baumannii* strains. Only 1 protein showed a percentage identity of <95% when compared to the proteome of *A. baumannii* ATCC 19606 using the BLASTp tool, for the final shortlist to contain 68 potential drug targets (Table S3).

**Figure 2:**
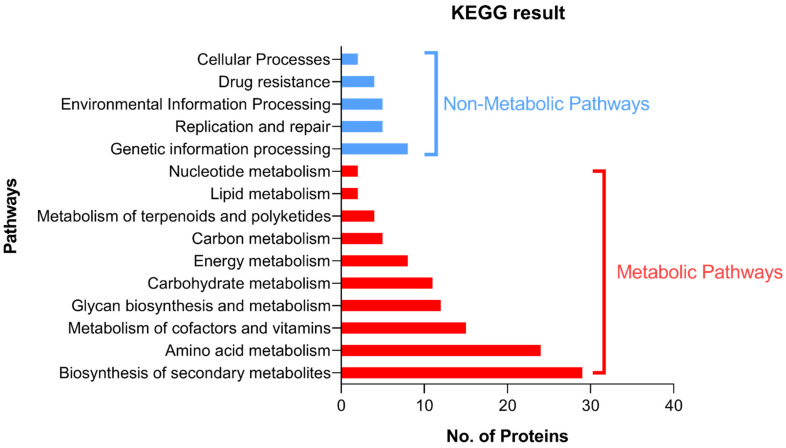
A diagram showing the distribution of the targeted proteins in different pathways. Blue bars represent the abundance of the proteins in different non-metabolic pathways and the red bars represent the abundance of the targeted proteins in different metabolic pathways.

For further demonstration of the pathway of the targeted proteins, KEGG was used to identify the pathways in which the shortlisted proteins are involved. Most of these proteins are involved in different metabolic pathways, where their distribution in each pathway is collectively demonstrated in Figure 2.

### 3.2. Testing the In Vitro Antimicrobial Activity of Candidate Ligands against A. baumannii 

**Figure 3:**
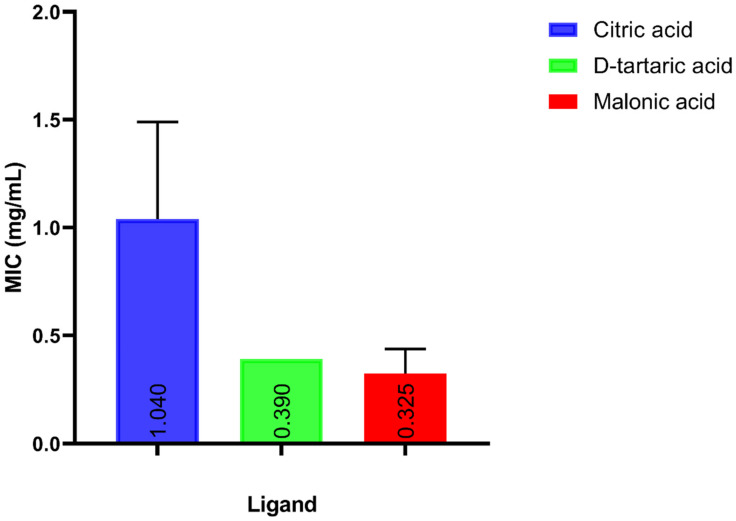
A bar chart representing the testing of the in vitro anti-acinetobacter activity of candidate ligands. The MIC of candidate ligands against *A. baumannii* ATCC 19606 was determined using the broth micro-dilution method. MIC values represent the mean of experimental triplicates. Error bars represent SD values.

Five ligands, citric acid, dipicolinic acid, D-tartaric acid, malonic acid and MES, which were expected to interact with glutamine synthetase, dihydrodipicolinate reductase (DHDPR), LpxA, aspartate 1-decarboxylase and bifunctional UDP-N-acetylglucosamine diphosphorylase/glucosamine-1-phosphate N-acetyltransferase (GlmU), respectively, were tested for their antibacterial activity against *A. baumannii*. The MIC of each of the candidate ligands against *A. baumannii* ATCC 19606 was determined using the broth micro-dilution method. Except for MES, the MIC values ranged from 325 to 1500 μg/mL, as shown in Figure 3. The MIC of dipicolinic acid was 1500 μg/mL, whereas that of MES was as high as 25 mg/mL.

### 3.3. Molecular Docking of Candidate Ligands to Their Target Proteins

#### 3.3.1. Docking of Citric Acid to Glutamine Synthetase

**Figure 4:**
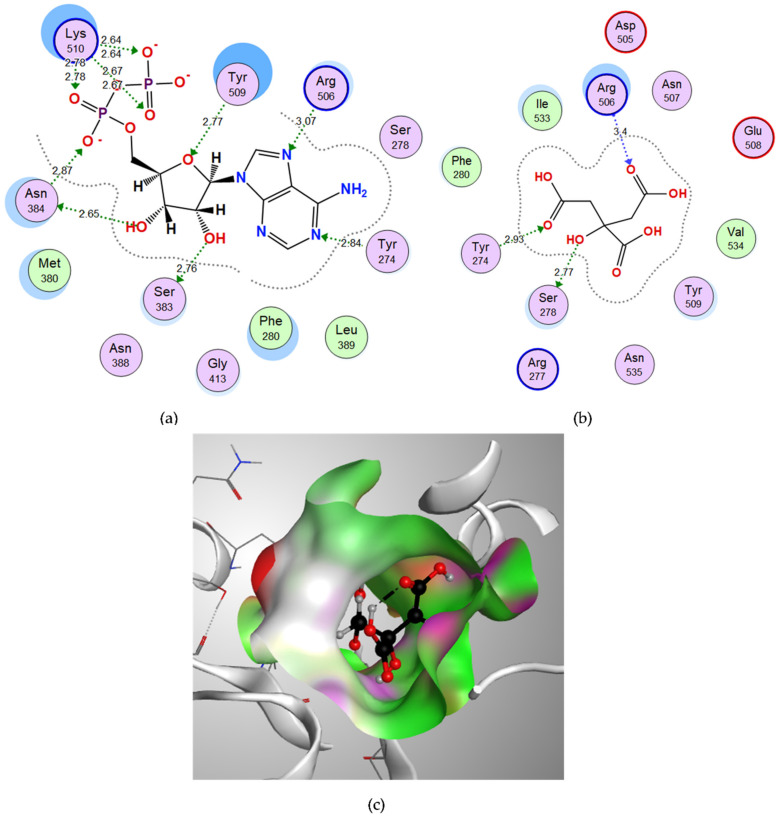
The molecular docking study of glutamine synthetase with the co-crystalized ligand adenosine diphosphate (ADP) and the candidate ligand citric acid: (**a**) Two-dimensional interactions of the co-crystalized ligand ADP within the glutamine synthetase active site; (**b**) Two-dimensional interactions of citric acid within the glutamine synthetase active site; (**c**) Three-dimensional interactions of citric acid within the glutamine synthetase active site.

Through the examination of the binding interactions of the co-crystalized ligand ADP, which was used to define the binding site for docking, it showed hydrogen bond interactions with Tyr274, Ser383, Asn384, Arg506, Tyr509 and Lys510, and the docking score (S) was −18.9321 kcal/mol. (Figure 4a). Citric acid showed a good binding energy score (−13.0519 kcal/mol.) and bound through strong H-bond interactions with its oxygen to Tyr274, Ser278 and Arg506 amino acids (Table 1). The 3D figure shows good fitting of citric acid in the binding site of glutamine synthetase (Figure 4c).

#### 3.3.2. Docking of Malonic Acid to Aspartate 1-Decarboxylase

**Figure 5:**
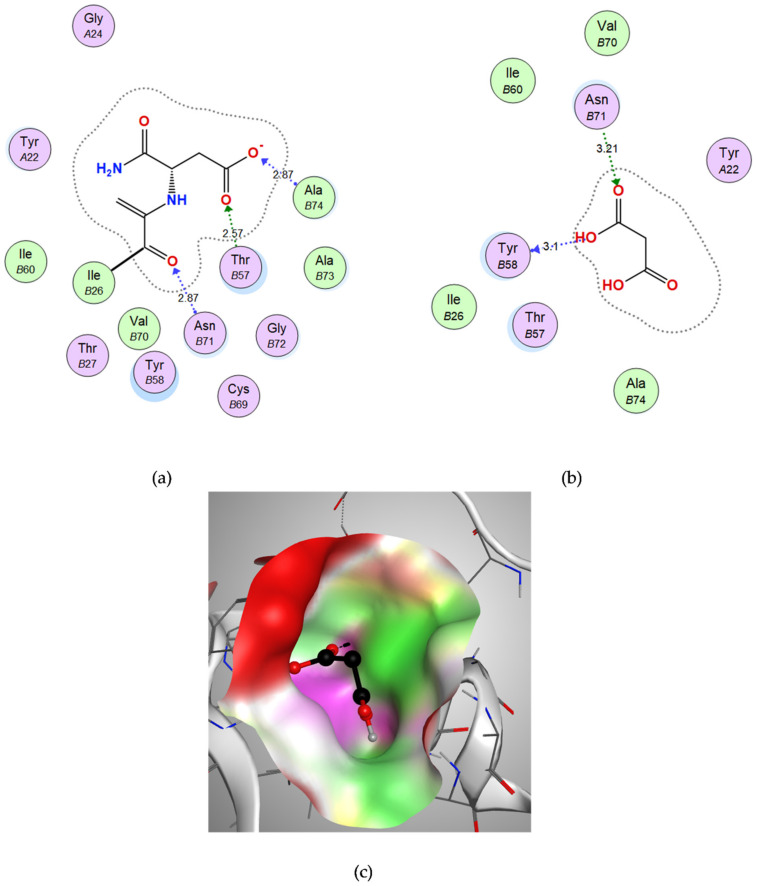
The molecular docking study of aspartate 1-decarboxylase with the co-crystalized ligand N~2~-(2-Amino-1-Methyl-2-Oxoethylidene)Asparaginate (NSN) and the candidate ligand malonic acid: (**a**) Two-dimensional interactions of the co-crystalized ligand NSN within the aspartate 1-decarboxylase active site; (**b**) Two-dimensional interactions of malonic acid within the aspartate 1-decarboxylase active site, (**c**) Three-dimensional interactions of malonic acid within the aspartate 1-decarboxylase active site.

The co-crystalized ligand NSN, which was used to define the binding site for docking, showed hydrogen bond interactions with chain B amino acids, Asn-B71, Ala-B74 and Thr-B57, with a binding energy score of −8.1205 kcal/mol. (Figure 5a). Malonic acid showed a good binding energy score (−8.5187 kcal/mol) and bound through strong H-bond interactions with its oxygen to Tyr-B58 and Asn-B71 amino acids (Table 1). The 3D figure reveals good fitting of malonic acid in the vicinity of the binding site of aspartate 1-decarboxylase (Figure 5c).

#### 3.3.3. Docking of D-Tartaric Acid to UDP-N-acetylglucosamine Acyltransferase

**Figure 6:**
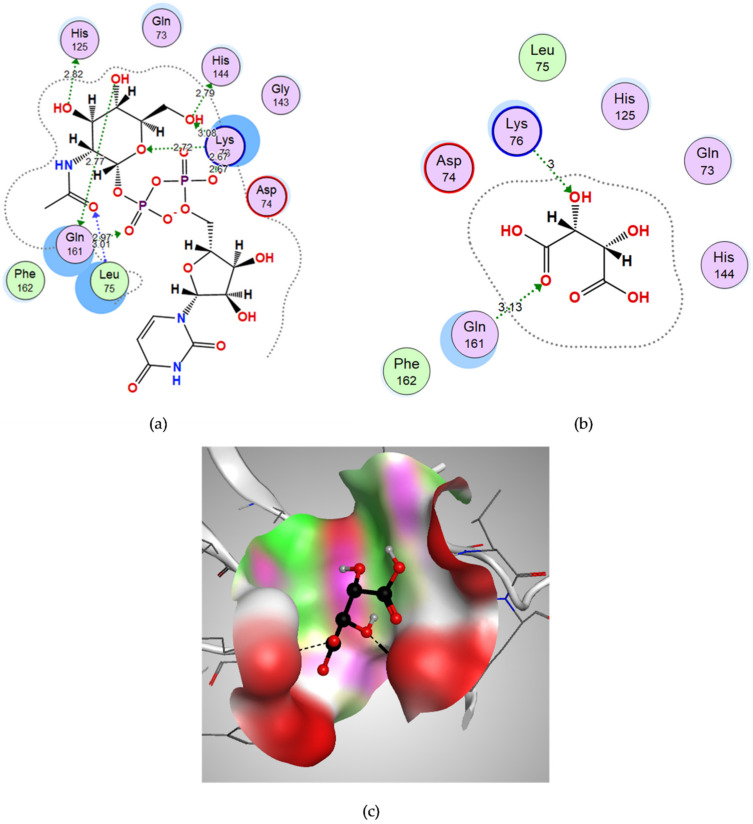
The molecular docking study of UDP-N-acetylglucosamine acyltransferase (LpxA) with the co-crystalized ligand Uridine-Diphosphate-N-Acetylglucosamine (UD1) and the candidate ligand D-tartaric acid: (**a**) Two-dimensional interactions of the co-crystalized ligand UD1 within the LpxA active site; (**b**) Two-dimensional interactions of D-tartaric acid within the LpxA active site; (**c**) Three-dimensional interactions of D-tartaric acid within the LpxA active site.

UD1 was used to define the binding site for docking, and it showed hydrogen bond interactions with Leu75, Lys76, His125, His144 and Gln161 with a binding energy score of −13.3676 kcal/mol. (Figure 6a). D-tartaric acid showed a good binding energy score (−9.2053 kcal/mol.) and bound through strong H-bond interactions with its oxygen to Lys76 and Gln161 amino acids (Table 1). The 3D figure shows the fitting of D-tartaric acid in the vicinity of the binding site of LpxA (Figure 6c). 

### 3.4. In Vivo Effect of Candidate Ligands on Wound Infection in the Mouse Model

**Figure 7:**
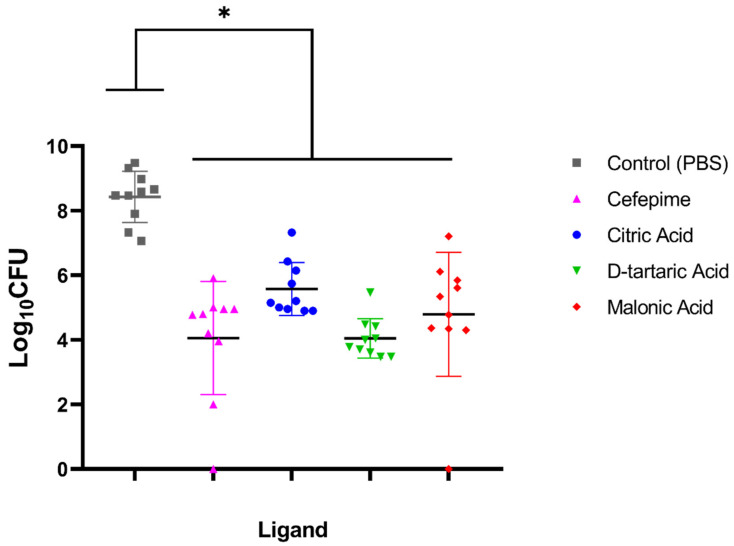
The antibacterial activity of citric acid, D-tartaric acid and malonic acid in comparison to cefepime and the control (PBS) against *A. baumannii* ATCC 19606 in a skin wound infection in the mouse model. Data are represented by the mean colony counts obtained from each of the five infected groups (*n* = 10 mice in each group) ± SD after six days of treatment. Statistical analysis was carried out using GraphPad, applying a one-way ANOVA followed by Tukey’s multiple comparisons test. (*) denotes a statistical significant difference (*p*-value < 0.0001) existing between groups.

Back-hair-shaved mice infected with *A. baumannii* ATCC 19606 were treated with the three ligands (citric acid, D-tartaric acid and malonic acid) to test the efficacy of the topical application of each of them on the wound infection (Figure 7). Comparisons of PBS as a control with each of the three ligands’ treatment of the wound infection revealed a reduction in colony forming units (CFU) per wound, which was statistically significant (*p*-value < 0.0001). On the other hand, the comparison of each of the three ligand treatments of the wound infection with the reference drug cefepime showed no significant difference (*p*-value = 0.0844, 0.714 and >0.9999 for citric acid, malonic acid and D-tartaric acid, respectively).

### 3.5. In Vitro Cytotoxicity Assay of Candidate Ligands against Human Skin Fibroblast

**Figure 8:**
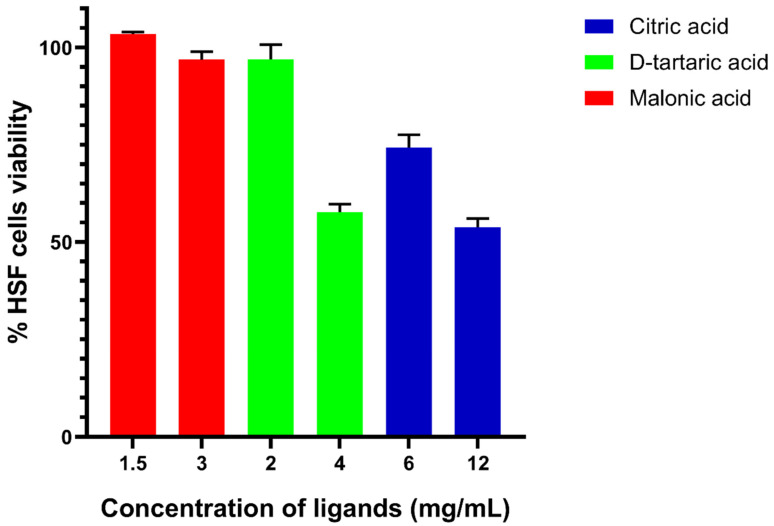
A bar chart representing the cytotoxicity of candidate ligands, citric acid, D-tartaric acid and malonic acid, against Human Skin Fibroblast (HSF) using a Sulforhodamine B (SRB) assay. Each candidate ligand was tested at two different concentrations equivalent to 5× and 10× its MIC against *A. baumannii* ATCC 19606. Results are presented as percent viable HSF cells. The values represent an average of three samples tested for each candidate ligand. Error bars represent SD values.

The cytotoxicity of the candidate ligands, citric acid, D-tartaric acid and malonic acid, was tested against HSF. The ligands showed good toxicity profiles against HSF (Figure 8). Malonic acid exhibited good tolerability even at higher concentrations (3 mg/mL) equivalent to 10× its MIC against *A. baumannii* ATCC 19606. D-tartaric acid and citric acid were tolerable to HSF at the concentrations equivalent to 5× their MIC of 2 and 6 mg/mL, respectively, and they showed reduced tolerability at higher concentrations, which represent 10× their MIC of 4 and 12 mg/mL, respectively.

### 3.6. In Vitro Confirmation of Aspartate 1-Decarboxylase Inhibition by Malonic Acid

**Figure 9:**
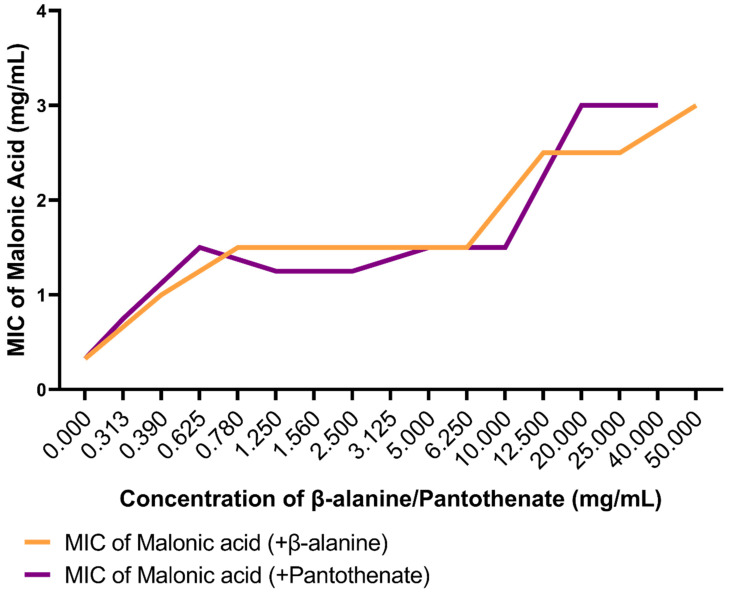
A chart representing the in vitro confirmation of aspartate 1-decarboxylase selective inhibition by malonic acid through showing the MIC of malonic acid against *A. baumannii* ATCC 19606 after the addition of increasing sub-inhibitory concentrations of the byproducts of the reaction catalyzed by aspartate 1-decarboxylase, β-alanine (orange line) and pantothenate (purple line).

Aspartate 1-decarboxylase is the enzyme responsible for the synthesis of β-alanine, which is further required in the biosynthesis of pantothenate. While concomitantly adding increasing sub-inhibitory concentrations of the byproducts of the reaction catalyzed by aspartate 1-decarboxylase, β-alanine (<200 mg/mL) and pantothenate (<250 mg/mL), the MIC values of malonic acid against *A. baumannii* ATCC 19606 were recorded in order to confirm its selective inhibitory action on aspartate 1-decarboxylase. The results show increasing MIC values of malonic acid from 0.325 mg/mL to 3 mg/mL (Figure 9).

**Figure 10:**
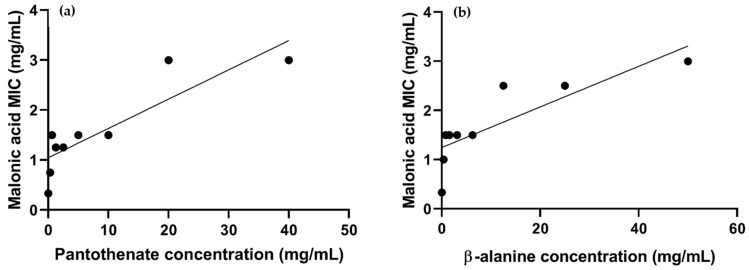
Charts showing the correlation between the MIC of malonic acid against *A. baumannii* ATCC 19606 and the increasing sub-inhibitory concentrations of the byproducts of the reaction catalyzed by aspartate 1-decarboxylase: (**a**) pantothenate (r = 0.8675); (**b**) β-alanine (r = 0.8313).

A strong uphill (positive) linear relationship was observed when testing the correlation between the increasing concentrations of pantothenate (r = 0.8675) or β-alanine (r = 0.8313), with the MIC of malonic acid (Figure 10).

## 4. Discussion

*Acinetobacter baumannii* infection has recently emerged as a serious issue associated with a high rate of mortality and morbidity. However, there is no permanent treatment for the drug-resistant *A. baumannii*, which is a case that calls for finding novel alternatives to commonly prescribed drugs with possibly different approaches. Approaches such as screening plant extracts [39,40,41], testing the efficiency of bacteriophages [42,43], a nano-particle-based approach [44,45] and an in silico virtual screening approach [46,47] have been used. 

In the current study, a subtractive proteomics approach was used to identify druggable targets for *A. baumannii*. The approach depends mainly on the successful passing of essential proteins through a number of channels to be considered finally as potential drug targets. First, essential proteins, which are necessary for the growth and survival of bacteria, grabbed our attention as effective drug targets [7]. To the best of our knowledge, this is the first time that OGEE was used to retrieve essential proteins. Most previous similar studies have used databases such as the Database of Essential Genes (DEG) and Geptop instead [12,13,15,17]. Avoiding possible undesired effects in human hosts is important, so essential proteins that are non-homologous to the human proteome were identified. Human-similar proteins were subtracted from essential proteins of the bacteria, which further prevented the cross-reactivity of drugs. The past two channels ensured mainly specificity and selectivity. Specificity was achieved by including essential proteins only, and selectivity was established by a human non-homology analysis. 

The next channel involved searching the druggability of the essential non-homologus proteins using DrugBank. DrugBank is a free-to-access, online database containing comprehensive information on drugs and drug targets. As both a cheminformatics and a bioinformatics resource, both detailed drug data and comprehensive drug target information are combined in this database. The latest release of DrugBank Online (version 5.1.9) contains 14,624 drug entries, including 2726 approved small molecule drugs, 1518 approved biologics, such as proteins, peptides, vaccines and allergenics, 132 nutraceuticals and over 6677 experimental drugs in the discovery phase. Additionally, 5274 non-redundant protein (i.e., drug target/enzyme/transporter/carrier) sequences are linked to these drug entries. Each entry contains more than 200 data fields with half of the information being related to drug data and the other half related to protein or drug target data [24]. In the current study, among the 351 essential human non-homologus proteins, 123 were found to have possible binding ligands in the DrugBank. These ligands were mostly experimental, where few were nutraceutical. Unlike most of the similar studies [16,17], the druggability channel was prioritized, as finding possible interacting ligands was a main aim of this study. 

The importance of the subcellular localization prediction was to depict the essential non-homologus proteins as drug or vaccine targets. Cytoplasmic proteins can be used for drug development, and membrane or secreted proteins, based on their antigenicity, can be used for vaccine development [48]. Therefore, cytoplasmic proteins, for which 105 were counted, were those of interest in our study. We preferred to obtain detailed information on the location of each essential non-homologus protein, even for those that were predicted with unknown localization using PSORTb. Therefore, another subcellular localization predictor CELLO was used.

It was necessary to confirm the conservation of these proteins in the available *A. baumannii* strains on NCBI to ensure the possible activity of the candidate drugs on all of them. The alignment of the potential proteins performed against the proteome of the *A. baumannii* ATCC 19606 was important, since these proteins primarily belong to the only available *A. baumannii* strain in OGEE, *A. baumannii* ATCC 17978, when retrieving the essential proteins in the first channel. 

Previous studies have reported identifying potential drug targets in *A. baumannii* by the subtractive proteomics approach [20,21,22,49]. Some of the 68 shortlisted drug targets in the current study have been reported in previous studies [20,21,22,50,51,52,53]. However, it is worth mentioning that our study is the first one to proceed with further in vitro and in vivo validation. 

Candidate drugs were initially selected after passing the constrictive channels (essentiality, non-homology with *Homo sapiens*, druggability, sub-cellular localization and conservation in *A. baumannii*) in our subtractive analysis along with the availability of these compounds in the market. Primarily, the activity of five candidate ligands was tested against *A. baumannii* ATCC 19606 through the determination of the MIC of each of them. The tested ligands, citric acid, dipicolinic acid, D-tartaric acid, malonic acid and MES, were supposed to interact with glutamine synthetase, DHDPR, LpxA, aspartate 1-decarboxylase and GlmU, respectively.

Glutamine synthetase is a necessary enzyme for the regulation of nitrogen metabolism and is used for the synthesis of glutamine via glutamate, ATP and ammonia. That is why ammonia is a special molecule for nitrogen anabolism in Gram-negative bacteria, whereas nitrogen is important for the synthesis of key elements of the cell, such as amino acids, NAD, pyrimidines, purines and amino sugars.

DHDPR catalyzes the second step of lysine biosynthesis. Lysine together with diaminopimelate are vital components of the bacterial peptidoglycan cell wall in both Gram-positive and Gram-negative bacteria. Therefore, lysine biosynthesis is known as a putative bacterial target [54]. 

LpxA initiates the lipid A biosynthetic pathway. Lipid A is the hydrophobic moiety that anchors the sugar contents (core and O-groups) of lipopolysaccharide (LPS) to the external surface of the outer membrane. Therefore, lipid A is essential for the viability of Gram-negative bacteria. Moreover, lipid A is an activator of the human immune system. LpxA has continuously been recognized as a potential target for antibacterial agents [55,56,57].

Aspartate 1-decarboxylase is involved in the biosynthesis of pantothenate (vitamin B5), which is further required for the synthesis of coenzyme A (CoA), a necessary molecule in energy metabolism that permits burning of carbohydrates, fats and proteins as energy sources. Aspartate 1-decarboxylase has been previously identified as a drug target in *Mycobacterium tuberculosis* [38,58].

The bifunctional enzyme GlmU is involved in the synthesis of UDP-N-acetylglucosamine, which is a key precursor in peptidoglycan, in Gram-negative bacteria and in LPS biosynthesis. The vital role of these elements in the maintenance of bacterial cell integrity and virulence have made GlmU an attractive target for antibacterial drug discovery. Inhibitors for GlmU in *Escherichia coli* and *Haemophilus influenzae* have been reported [59,60].

Upon testing the MIC, malonic acid had the best anti-acinetobacter activity among the ligands in the current study (MIC = 325 μg/mL), and MES showed the least anti-acinetobacter activity (MIC = 25 mg/mL). Apart from MES, the MIC values of the candidate drugs in the study varied from 325 to 1500 μg/mL.

All the selected ligands shared the characteristic of being non-antibiotic agents. Various previous studies have discussed the antibacterial activity of non-antibiotic agents [61,62,63,64,65,66]. In these studies, the antibacterial activity of NSAIDs and statins against *A. baumannii*, in addition to other Gram-negative rods, has been reported [61,66]. Interestingly, the MIC values reported in these studies are comparable to the values in the current study. For example, diclofenac showed direct antibacterial activity against *Proteus vulgaris* NCTC 4635, *Proteus mirabilis* ATCC 12453, *Burkholderia cepacia* ATCC 45216 and *Stenotrophomonas maltophilia* ATCC 12714, with the MIC ranging from 200 to 800 μg/mL, and its MIC against *A. baumannii* ATCC 19606 was 1600 μg/mL [61]. Moreover, rosuvastatin exhibited antibacterial activity against a group of both Gram-positive and Gram-negative bacteria, with the MIC ranging from 104.17 μg/mL to 500 μg/mL and with an MIC = 333.33 μg/mL against *A. baumannii* ATCC 17978 [66].

From the selected candidate drugs, citric acid, D-tartaric acid and malonic acid share one more characteristic of being short-chain organic acids (C₆H₈O₇, C_4_H_6_O_6_ and C_3_H_4_O_4_). The antibacterial activity of these organic acids has been previously reported in several studies [67,68,69]. It has been reported that citric acid exhibits antibacterial activity against *Helicobacter pylori* and *Pseudomonas aeruginosa* [12,68,70]. Over et al. reported the antibacterial activity of some organic acids, including both citric acid and tartaric acid on *Escherichia coli* O157:H7, *Listeria monocytogenes* and *Salmonella typhimurium* [71]. Elisa et al. reported the antibacterial activity of tartaric acid against *Campylobacter* spp., with the MIC ranging from 8 to 256 mmol/L [72]. Moreover, the antibacterial activity of malonic acid was reported as one of the components of pine needle aqueous extract, which had activity against *Bacillus subtilis*, *Staphylococcus aureus*, *Bacillus cereus*, *Micrococcus luteus*, *Escherichia coli*, and *Proteus vulgaris* [73]. Moreover, organic acids have increasingly been used in veterinary medicine for their antimicrobial properties [74,75,76]. In addition, previous studies have noted the role of organic acids in combating foodborne illness [69,71,72,77]. Regarding safety, organic acids have a well-documented history of being safely used as food preservatives [78].

To the best of our knowledge, the current study is the first one reporting the antibacterial activity of the selected ligands on *A. baumannii*.

In light of the in vitro study, the ligands with the lowest MIC values (citric acid, D-tartaric acid and malonic acid) were further tested to investigate if their antibacterial activity could be maintained in the murine wound infection model by *A. baumannii*. This model was selected based on the known information that wound infections are one of the most significant infections caused by *A. baumannii* [79]. Several studies have shown the applicability of topically applied non-antibiotic agents in the treatment of *A. baumannii* wound infections [32,80,81,82].

The three tested ligands, citric acid, D-tartaric acid and malonic acid, showed a significant reduction in the bacterial load than that observed in the control group treated with PBS. Unexpectedly, D-tartaric acid showed the highest reduction in the bacterial load. It was noted that, although none of the tested ligands showed a significant difference from the reference drug cefepime, both D-tartaric acid and malonic acid showed a comparable efficacy to that of cefepime. Cefepime was considered as a reference drug, since it is regarded as a drug of choice for the treatment of *A. baumannii* infections [83].

Although no visible skin injuries appeared upon applying any of the three ligands (citric acid, D-tartaric acid and malonic acid) in the performed mouse model, it was important to test the cytotoxicity of the three ligands to ensure avoiding any possible harmful effects on host tissues. Malonic acid showed the best cytotoxicity profile against HSF, where about 100% of the cells remained viable at both concentrations equivalent to 5× and 10× the MIC of malonic acid. It was noted that both citric acid and D-tartaric acid exhibited relatively reduced tolerability at higher concentrations equivalent to 10× the MIC of each of them. Although the HSF cell viability exceeded 50%, it can be improved by reducing the therapeutic dose in further in vivo studies.

From our findings, it is clear that malonic acid proved to be the most promising ligand. An additional confirmatory test was performed for the selective inhibition of malonic acid to its target protein, aspartate 1-decarboxylase. The MIC of malonic acid showed a dose-dependent escalation by the exogenous supply of increasing sub-inhibitory concentrations of both β-alanine and pantothenate, the downstream products of the aspartate 1-decarboxylase catalyzed reaction. The results are also represented by a strong uphill (positive) linear relationship for the test of the correlation between the increasing concentrations of pantothenate (r = 0.8675) or β-alanine (r = 0.8313) with the MIC of malonic acid. Similar to our findings, pantothenate and β-alanine antagonized the activity of pyrazinamide, a first-line anti-tuberculosis drug targeting aspartate 1-decarboxylase, suggesting the selective targeting of the drug to the enzyme involved in pantothenate biosynthesis [38,84].

## 5. Conclusions

Applying the subtractive proteomics approach in the current study revealed 68 potential druggable targets in MDR *A. baumannii*. Out of these targets, 3 targets (aspartate 1-decarboxylase, glutamine synthetase and LpxA) have experimentally—both in vitro and in vivo—proven to be lethal to *A. baumannii* by the effect of interacting ligands (malonic acid, citric acid and D-tartaric acid). Due to the promising anti-acinetobacter activity of these ligands with good safety profiles, we propose them to be the subjects of future investigations for the treatment of *A. baumannii* infections. Moreover, the rest of the targets may serve as potential candidates for upcoming in-depth validation studies.

## Figures and Tables

**Table 1:** Summary of the docking results of the binding of candidate ligands Citric acid, Malonic acid and D-tartaric acid to target proteins Glutamine Synthetase, Aspartate 1-Decarboxylase and UDP-N-acetylglucosamine Acyltransferase, respectively.

Compound	Binding Score (kcal/mol)	Amino Acids at Active Site	Interacting Groups of Ligand	Type of Interaction	Bond Length
Citric acid	−13.0519	Tyr274	O (C=O)	H-bond acceptor	2.93
		Ser278	OH	H-bond donor	2.77
		Arg506	O (C=O)	H-bond acceptor	3.40
Malonic acid	−8.5187	Tyr-B58	OH	H-bond donor	3.10
		Asn-B71	O (C=O)	H-bond acceptor	3.21
D-tartaric acid	−9.2053	Lys76	OH	H-bond acceptor	3.00
		Gln161	O (C=O)	H-bond acceptor	3.13

## Data Availability

Data will be available upon request.

## Supplementary Materials

The following supporting information can be downloaded at: https://www.mdpi.com/article/10.3390/microorganisms10101973/s1, Table S1: Characteristics of proteins retrieved from the OGEE database and the flow of the subtractive analysis through each channel, Table S2: Available *Acinetobacter baumannii* strains in the NCBI database checked for the conservation of target proteins, Table S3: Shortlisted protein drug targets that passed all the subtractive channels with their possible binding ligands.
